# Differences in post-disaster mental health among Vietnamese and African Americans living in adjacent urban communities flooded by Katrina

**DOI:** 10.1371/journal.pone.0255303

**Published:** 2021-08-25

**Authors:** Mengxi Zhang, Mark VanLandingham, Yoon Soo Park, Philip Anglewicz, David M. Abramson

**Affiliations:** 1 Department of Nutrition and Health Science, School of Health, Ball State University, Muncie, Indiana, United States of America; 2 Department of Social, Behavioral, and Population Sciences, School of Public Health and Tropical Medicine, Tulane University, New Orleans, Louisiana, United States of America; 3 Massachusetts General Hospital and Harvard Medical School, Boston, Massachusetts, United States of America; 4 Department of Population, Family and Reproductive Health, School of Public Health, Johns Hopkins Bloomberg University, Baltimore, Maryland, United States of America; 5 School of Global Public Health, New York University, New York, New York, United States of America; Drexel University, UNITED STATES

## Abstract

Some communities recover more quickly after a disaster than others. Some differentials in recovery are explained by variation in the level of disaster-related community damage and differences in pre-disaster community characteristics, e.g., the quality of housing stock. But distinct communities that are similar on the above characteristics may experience different recovery trajectories, and, if so, these different trajectories must be due to more subtle differences among them. Our principal objective is to assess short-term and long-term post-disaster mental health for Vietnamese and African Americans living in two adjacent communities in eastern New Orleans that were similarly flooded by Hurricane Katrina. We employ data from two population-based cohort studies that include a sample of African American adults (the Gulf Coast Child and Family Health [GCAFH study]) and a sample of Vietnamese American adults (Katrina Impacts on Vietnamese Americans [KATIVA NOLA study]) living in adjacent neighborhoods in eastern New Orleans who were assessed near the second and thirteenth anniversaries of the disaster. Using the 12-Item Short Form Survey (SF-12) as the basis of our outcome measure, we find in multivariate analysis a significant advantage in post-disaster mental health for Vietnamese Americans over their African American counterparts at the two-year mark, but that this advantage had disappeared by the thirteenth anniversary of the Katrina disaster.

## Introduction

### Background

Disasters affect health and well-being in a multitude of ways [1], including mental health [2–6]. In addition to mental health challenges that appear shortly after disasters, many people experience long-term impacts [7–9]. Not all subgroups will experience these problems to the same degree or in the same way [4]. Soon after Hurricane Katrina, African Americans reported greater levels of stress than Whites [10]. Soon after Hurricane Andrew, Spanish-speaking Latinos showed a higher rate of post-traumatic stress disorder (PTSD) compared with African Americans and Whites [11]. Fourteen years after the Buffalo Creek dam collapse, African Americans were more likely to show delayed PTSD than were Whites [12]. Minorities are not always found to be disadvantaged post-disaster. For example, Vietnamese Americans showed much lower rates of PTSD in the aftermath of Hurricane Katrina than did similarly affected groups [13].

Many of these inter-group differences in post-disaster mental health status can be explained by systematic differences in exposure across groups. Families of the same ethnicity often cluster together in communities, especially within cities. These urban communities often have vast differences in exposure, *e*.*g*., for flooding depth. This raises the question: how would two very different urban ethnic communities fare if they were contiguous, had similar housing stock, and experienced the same levels of flooding and damage?

### Theoretical and conceptual framework

Disaster researchers generally attribute disparities in post-disaster recovery among communities to differences in the degree of impact and differences in pre-disaster characteristics that predispose some communities to recover more quickly and completely than others. To assess recovery, researchers usually focus on one or more of the following dimensions: mental health, physical health, housing stability, economic stability, and social role adaptation [14]. We focus on mental health here because good post-disaster mental health implies a return to “routine, normalcy, or stability;” not surprisingly, mental health has one of the strongest associations with an underlying latent measure of general recovery [14]. The set of pre-disaster characteristics that predispose some communities to recover more quickly and completely than others is conceptualized by many disaster scholars as “resilience.” Community resilience typically encompasses a set of attributes that are linked to “a positive trajectory of functioning and adaptation after a disturbance” [15]. In the framework developed by Fran Norris and her colleagues, these attributes include economic development, social capital, information and communication, and community competence [15].

Our “natural experiment” research design for the current analysis exploits the fact that the African American and Vietnamese communities that we consider here had nearly identical levels of storm-related impact from Hurricane Katrina, and were subsequently assessed with the same measures post-Katrina at close to the same points in time. Therefore, any disparities in post-Katrina recovery should be attributable to factors that are distinct from levels of impact, that is, they must be attributable to differences in resilience. We consider ethnicity to be a proxy variable that should capture the distinct packages of resilience for these two distinct—but adjacent—communities.

## Methods

### Participants

A major advantage of our approach is the availability of post-Katrina recovery data for Vietnamese Americans and African Americans who were living in adjacent and very similar neighborhoods in eastern New Orleans at the time of Hurricane Katrina and afterward. These data are from two population-based cohort studies on the long-term effects of Hurricane Katrina on health and well-being. The first study consists of only Vietnamese Americans and is called the Katrina Impacts on Vietnamese Americans (KATIVA NOLA) study. The second, the Gulf Coast Child and Family Health (GCAFH) study consists of an equal mix of African American and white residents of Katrina-exposed regions. Informed consent (written or verbal, per the participant’s wishes) was obtained for all KATIVA NOLA participants per procedures approved by Tulane University’s IRB. Tulane University Human Research Protection Office approved the KATIVA NOLA arm of the study [(IRB#: 218101]. Columbia University Medical Center IRB approved the original GCAFH study, IRB-AAAB8668 (2/11/2006), with a waiver of written informed consent. New York University IRB approved the continuing GCAPH study, IRB-FY2016-274, also with a waiver of written informed consent.

The KATIVA NOLA study is a longitudinal study that measures the impact of Hurricane Katrina on the Vietnamese American community in the greater New Orleans area. The initial wave was designed as a study of immigrant health and was conducted a few weeks before Katrina struck in the late summer of 2005. Follow-up waves focused on the impact of and recovery from Katrina, and were conducted in 2006, 2007, 2010, and 2018. Respondents are first-generation working-age Vietnamese Americans living in the greater New Orleans area; most reside in the principal enclave in eastern New Orleans. Respondents were randomly selected from a comprehensive list of all Vietnamese American families living in the New Orleans metropolitan area that was updated just before Hurricane Katrina struck in the summer of 2005; the list was maintained by the principal church and non-government organizations serving this community. For households that included more than one eligible respondent, a random selection procedure was employed to select one. A total of 125 participants were interviewed during the summer of 2005; 91 (72.8%) and 86 (68.8%) of these were re-interviewed near the two-year and thirteen-year anniversary of the disaster in 2007 and 2018, respectively. Among those, 70 (76.9%) and 59 (68.6%) lived in the principal Vietnamese enclave in eastern New Orleans in the following waves. This is the Vietnamese American sub-sample used in the analyses for this paper.

The cohort for GCAFH was enrolled in Louisiana in February 2006 and in Mississippi in August 2006 using a stratified cluster sampling design, employing lists of residents in congregate housing sites (Federal Emergency Management Agency (FEMA) group sites, commercial trailer sites, and hotels) obtained from FEMA. The waves employed for this analysis were collected in 2007 and 2018, near the second and thirteenth anniversary of Hurricane Katrina. Among the 1,079 respondents interviewed in the first wave, 803 (74.2%) and 647 (59.8%) were re-interviewed in 2007 and 2018. Among those, 74 and 50 working-age African Americans were living in communities adjacent to the principal Vietnamese enclave in eastern New Orleans in 2007 and 2018, respectively. This is the African American sub-sample used in the analyses for this paper.

In total, 124 (2007:74; 2018:50) African American interviews and 129 (2007:70; 2018:59) Vietnamese American interviews are included in the analysis. Not everyone was re-interviewed in both the 2007 and 2018 waves. Among African Americans, 33.8% (25/74) were re-interviewed in both waves. Among Vietnamese Americans, 69.9% (51/70) were re-interviewed in both waves.

### Measures

#### Outcome

KATIVA NOLA and GCAFH measured key outcomes and predictors using the same instruments. Mental health status was measured in both studies using the mental component score (MCS) of the Short Form 12 Health Survey Questionnaire SF-12 (SF-12). SF-12 is a 12-item questionnaire that is used to measure overall general physical and mental health [16]. The index of MCS ranges from 0 to 100 with the higher scores representing better mental health [16]. SF-12 has been translated into many languages and widely used among various populations, including Vietnamese and Vietnamese Americans [17–19].

#### Covariates

In many ways, the contiguous and similar neighborhoods provide a “natural experiment” that controls for important potentially confounding factors that exist at the neighborhood level, such as housing stock, investments by local, state, and national government, as well as housing damage from Katrina. In addition to these built-in controls, both studies include measures of important background characteristics measured at the individual and household level that could confound our principal relationship of interest, *i*.*e*., the relationship between ethnicity (or race) and post-disaster mental health. Such background measures include age, sex, marital status, homeownership, and employment status. Sex was coded as a binary variable with 1 (male) or 0 (female). Age in years was a continuous variable. Marital status was coded as a binary variable with 1 (Married) or 0 (Single/previous married). Homeownership and employment were binary variables with 1 (Yes) or 0 (No). We tested other measures including education, car ownership, duration of residence, and return to pre-Katrina address, but none of these measures were significantly associated with mental health in our regression models and did not improve the overall model fit. Therefore, we did not include these variables in the adjusted models.

#### Data analysis

First, we identified bivariate differences in characteristics between African Americans and Vietnamese Americans in 2007 and 2018. Categorical variables were compared using chi-square tests and Fisher’s exact test if any cell has expected frequencies less than 5; continuous variables were compared using two-sample t-tests. To measure the association between ethnicity and post-disaster mental health recovery, we first compared the mental health status between the two ethnic groups in each wave, applying crude and adjusted linear regression models. Covariates included in the adjusted linear regression model are age, sex, marital status, homeownership, and employment status; these covariates are known to be associated with mental health from related studies [20, 21].

We then compared the mental health status between the two ethnic groups using a combined two waves unbalanced panel dataset. We fit three generalized estimating equations (GEE) to measure population-averaged effects of ethnicity on mental health [22]. GEE from the Gaussian distribution was used to account for the correlated structure of the repeated measures in 2007 and 2018. In the first model, we compared post-disaster mental health status between the two ethnic groups by adjusting year, age, sex, marital status, homeownership, and employment status. In model two, we compared the changes in post-disaster mental health for African Americans and Vietnamese Americans between the two waves by including an interaction term between year and ethnicity. We also tested interactions between year and race, sex, marriage, homeownership, and employment in order to examine whether the relationship between these covariates and mental health changed over time (only the interaction between employment and year was significant and included in the third model).

Due to the small sample sizes available for analysis, we employed a significance level of 0.10 for our two tailed tests of statistical significance. Data analysis was conducted using Stata/SE15.1.

## Results

Table 1 shows bivariate differences in the characteristics between the two samples, separately in the two waves. More Vietnamese American males than females were involved in the analysis, while more African American females than males were included. The large gender imbalance among Vietnamese Americans is due to the fact that more men than women left Vietnam in the aftermath of what is known in the U.S. as the Vietnam War [23]. The mean age for our Vietnamese American sample was about four years older than the mean age for the African Americans in 2007. Vietnamese Americans had an advantage in social-economic status, including employment and homeownership in 2007. Though the advantage in homeownership still existed in 2018, all these indicators improved significantly for African Americans between 2007 and 2018.

**Table 1:** Background characteristics of the African American and Vietnamese American participants in 2007 and 2018, applying bivariate analysis.

Variable	2007			2018		
	African Americans	Vietnamese Americans	*p* value	African Americans	Vietnamese Americans	*p* value
**Sex,%**						
Female	60.81	32.86	0.001^a^	64.00	40.68	0.015 ^a^
Male	39.19	67.14		36.00	59.32	
**Marital status,%**						
Single/ Previous married	63.51	13.04	<0.001 ^a^	66.00	20.34	< 0.001 ^a^
Married	36.49	86.96		34.00	79.66	
**Homeownership,%**						
No	56.76	21.43	<0.001^a^	40.00	10.34	< 0.001 ^a^
Yes	43.24	78.57		60.00	89.66	
**Employed,%**						
Unemployed	31.43	7.89	< 0.001^a^	16.00	11.86	0.532 ^a^
Employed	68.57	92.11		84.00	88.1	
**Age (years), Mean (SD)**	41.47 (10.97)	45.57 (4.89)	0.003 ^b^	55.28 (12.40)	55.27 (4.60)	0.498 ^b^
**Physical Health (scores), Mean (SD)**	48.22 (11.67)	50.69 (9.17)	0.081^b^	49.30 (11.17)	48.27 (8.07)	0.291 ^b^
**Mental Health (scores), Mean (SD)**	41.35 (13.84)	47.61 (11.93)	0.002^b^	48.88 (1.72)	52.78 (9.09)	0.030 ^b^
**Number**	74	70		50	59	

^a^ Chi-square test.

^b^ Two-sample t-test.

^c^ Fisher’s exact test.

Some differences in characteristics between the two groups were maintained across waves. In addition to the gender differences noted above, Vietnamese Americans were more than twice as likely to be married as African Americans. Regarding our major outcome variable of interest, before taking into account any of these differences in the composition of the two samples, Vietnamese Americans have significantly better mental health status than do the African American respondents in both waves. Both groups experienced an improvement in their mental health from 2007 to 2018, but the African Americans (7.35) experienced greater improvement compared with the Vietnamese American respondents (5.17).

Table 2 takes these compositional differences between the two samples into account in our linear regression models for post-disaster mental health status in 2007 and 2018, applying crude and adjusted linear regression models. The crude model shows an advantage in mental health status for the Vietnamese Americans over the African Americans by 6.26 and 3.90 points in 2007 and 2018, respectively. After controlling for covariates, the adjusted model shows that the advantage in mental health among the Vietnamese Americans is maintained in 2007 (Coefficient 5.31; 95% CI -0.02, 10.65), while this advantage became insignificant by 2018 (Coefficient 1.13; 95% CI -3.28, 5.54). Additionally, younger age (Coefficient -0.22; 95% CI -0.48, 0.04) in 2007 as well as being married (Coefficient 4.06; 95% CI -0.67, 8.78) or employed (Coefficient 9.32; 95% CI 3.80, 14.84) in 2018 were protective factors for post-disaster mental health recovery.

**Table 2:** Differences of post-disaster mental health between African American and Vietnamese American participants in 2007 (N = 144) and 2018 (N = 109), applying linear regression models.

Variable	2007		2018	
	Crude Model	Adjusted Model	Crude Model	Adjusted Model
	Coefficient (95% CI)	Coefficient (95% CI)	Coefficient (95% CI)	Coefficient (95% CI)
**Ethnicity**				
African Americans (ref.)				
Vietnamese Americans	6.26*** (1.99, 10.53)	5.31* (-0.02, 10.65)	3.90 (-0.15, 7.95)	1.13 (-3.28, 5.54)
**Age**		-0.22* (-0.48, 0.04)		0.14 (-0.08, 0.36)
**Sex**				
Female (ref.)				
Male		-0.57 (-5.11, 3.97)		2.51 (-1.41, 6.43)
**Marital status**				
Single/ Previous married (ref.)				
Married		4.36 (-1.12, 9.82)		4.06* (-0.67, 8.78)
**Homeownership**				
No (ref.)				
Yes		1.94 (-3.16, 7.05)		0.22 (-5.00, 5.44)
**Employment**				
No (ref.)				
Yes		-3.07 (-9.05, 2.91)		9.32*** (3.80, 14.84)

SE: Standard Error; 95% CI: 95% Confidence Interval; Significance:

* p<0.1

** p<0.05,

*** p<0.01.

Covariates included in the adjusted linear regression models are age, sex, marital status, homeownership, and employment status.

Table 3 presents comparisons of overall post-disaster mental health status between the two ethnic groups using a combined two-wave dataset, applying three GEE models. In model one, Vietnamese Americans did not have significantly better mental health (Coefficient 2.40; 95% CI-0.79, 5.59), after controlling for the impacts from year, age, sex, marital status, homeownership, and employment status. There were again no statistically significant differences in mental health between groups after including an interaction term with year and ethnicity (Coefficient 3.11; 95% CI -1.47, 7.70). Because employment status is important in 2018 but not in 2007 (see Table 2), we included in Model 3 an additional interaction term between year and employment. After including this term, the difference in mental health between groups was statistically significant (Coefficient 4.60; 95% CI -0.17, 9.36). Those who were married had significantly better mental health compared with those who were not in all three models (model 1: Coefficient 3.64; 95% CI 0.07, 7.21; model 2: Coefficient 3.65; 95% CI 0.09, 7.21; model 3: Coefficient 3.94; 95% CI 0.25; 7.62). The results for the interaction term show that those who were employed by 2018 had significantly better mental health than the reference group. The measure for ethnicity in this model shows that when both African Americans and Vietnamese Americans are unemployed, Vietnamese Americans have significantly better mental health.

**Table 3:** Differences of post-disaster mental health between African American and Vietnamese American participants using a combined 2007 and 2018 dataset (N = 253), applying generalized estimating equations models.

Variable	Model one	Model two	Model three
	Coefficient (95% CI)	Coefficient (95% CI)	Coefficient (95% CI)
**Ethnicity**			
African Americans (ref.)			
Vietnamese Americans	2.40 (-0.79, 5.59)	3.11 (-1.47, 7.70)	4.60* (-0.17, 9.36)
**Year**			
2007 (ref.)			
2018	6.15*** (2.48, 9.82)	7.01** (1.33, 12.70)	-2.78 (-10.41, 4.85)
**Age**	-0.02 (-.21, 0.17)	-0.03 (-0.22, 0.16)	-0.06 (-0.25, 0.13)
**Sex**			
Female (ref.)			
Male	0.85 (-1.95, 3.80)	0.83 (-2.17, 3.84)	0.76 (-2.22, 3.73)
**Marital status**			
Single/ Previous married (ref.)			
Married	3.64 ** (0.07, 7.21)	3.65** (0.09, 7.21)	3.94** (0.25, 7.62)
**Homeownership**			
No (ref.)			
Yes	1.60 (-2.09, 5.30)	1.62 (-2.08, 7.31)	1.57 (-2.18, 5.33)
**Employment**			
No (ref.)			
Yes	2.68 (-1.85, 7.22)	2.52 (-2.13, 7.17)	-2.94 (-8.79, 2.90)
Year*Race		-1.57 (-7.30, 4.17)	-3.48 (-9.30, 2.35)
Year*Employment			13.20*** (4.80, 21.60)

SE: Standard Error; 95% CI: 95% Confidence Interval; Significance:

* p<0.1

** p<0.05,

*** p<0.01.

Covariates included in model one are year, age, sex, marital status, homeownership, and employment status.

Covariates included in model two are year, age, sex, marital status, homeownership, employment status, and an interaction term between year and ethnicity.

Covariates included in model two are year, age, sex, marital status, homeownership, employment status, an interaction term between year and ethnicity, and an interaction term between year and employment.

## Discussion

Systematic differences in recovery across communities characterize most disasters. Much of this variation has been attributed to corresponding differences in the degree of disaster-related impact but sometimes similarly-impacted communities recover differently. Post-disaster mental health assessments from two distinct ethnic communities that were contiguous, that experienced very similar amounts of flooding, that were similar in pre-disaster housing stock, and that were assessed at the same two time points within a multivariate framework provide a rare opportunity to explore our central question: might ethnic differences in post-disaster well-being emerge and persist across these two comparable settings?

The answer is yes (for emerge) and no (for persist). Multivariate models indicate a substantial and statistically significant advantage in post-disaster mental health at around the two-year anniversary of the event for Vietnamese Americans *vis a vis* their African American counterparts. Our descriptive statistics show that between the second and thirteenth years after Katrina, the average mental health scores for both groups improved, but the improvement was more marked for African Americans. By the thirteenth anniversary of Katrina in 2018, the advantage for Vietnamese Americans had evaporated in our multivariate models. At this later wave, the two statistically significant predictors are marriage and employment status. The marriage effect seems straightforward. It is positive and is about the same size in 2007 and 2018, but it is only significant in 2018. As expected, being married seems to confer a mental health advantage with respect to the unmarried, likely due to both the benefits of being married (*e*.*g*., companionship, the possibility of dual incomes, shared work, *etc*.) and selection effects (*i*.*e*., the unmarried may suffer from disadvantages that both limit opportunities for marriage and negatively affect mental health status).

The employment effect seems more complex. Our descriptive statistics show that while employment levels were very steady for the Vietnamese across the two waves, for African Americans employment rose from 69% of the sample in 2007 to 84% in 2018 (homeownership improved, too). The effect of being employed (or unemployed) changed a lot across the two waves, too. From our multivariate models it is clear that for anyone finding themselves unemployed in 2018, the penalty for mental health status was substantial indeed.

An alternative treatment of the data that includes interaction effects and that employs a GEE framework to account for the correlated structure of the repeated measures in the two waves as well as differences in sample composition related to age, sex, employment, and marital and socioeconomic status produces several results that are similar to those from the linear regressions described above. Vietnamese Americans have better mental health than African Americans at the earlier (2007) post-Katrina wave and unemployment severely dampens mental health status at the later (2018) wave for both Vietnamese American and African American respondents (we tested for an interaction between ethnicity and employment and it is not statistically significant).

Viewing the results in their entirety, African Americans face disadvantages in mental health in the short term (2 years) aftermath of Hurricane Katrina, but this ethnic disadvantage disappears in the longer term (13 years) only to be replaced by an important role for employment.

We offer two complementary possible explanations. The African American disadvantage in mental health in 2007 is driven largely by poor employment prospects available for them at that time. In related work, some of us have found that during the early resettlement of New Orleans after Hurricane Katrina, African Americans were displaced for significantly longer periods than were whites or Vietnamese Americans, leaving them seeking work in an unfamiliar environment for longer periods of time [24]. In other related work, some of us have reported evidence for clear preferences for hiring Vietnamese Americans in the immediate aftermath of Katrina by local employers [25]. Although comparisons to African Americans are not explicit, they are often clearly implied. Distinct features of the African American and Vietnamese American communities that influence the pace of return and negative and positive stereotypes about these communities are in turn influenced by broader social forces related to history and culture [26]. Conventional frameworks of community resilience cover well the importance of employment as a form of “economic development” [15], but, as one of us has argued elsewhere, are woefully inadequate in their treatment of “upstream variables” related to history and culture [25].

Regarding policy, programs that facilitate employment for African Americans and other disadvantaged minorities soon after a disaster may hasten the recovery of mental health. Addressing the upstream variables that result in a wide range of enduring disadvantages for African Americans requires fundamental social change.

Regarding limitations of the study, an ideal study would benefit from a very large probability sample of all of Eastern New Orleans that would include a representative sample of both African Americans and Vietnamese Americans; and would include multiple waves both before and after Katrina with high retention rates. However, no such data exist. Given the importance of the research questions, we improvise using high quality data assessing both populations of interest that happen to live next to each other (providing a set of built in controls), had very similar flooding damage from Katrina, and were assessed in two waves at around the same time. This approach mitigates the inherent limitations of our small samples and the methodological challenges of merging data from two different studies.

## Supporting information

S1 Data (DTA)
